# Embodied health: the effects of a mind–body course for medical students

**DOI:** 10.3402/meo.v18i0.20699

**Published:** 2013-04-30

**Authors:** Allison R. Bond, Heather F. Mason, Chelsey M. Lemaster, Stephanie E. Shaw, Caroline S. Mullin, Emily A. Holick, Robert B. Saper

**Affiliations:** 1 Boston University School of Medicine, Boston, MA, USA; 2 Program for Integrative Medicine and Health Care Disparities, Department of Family Medicine, Boston University School of Medicine, Boston, MA, USA; 3 The Minded Institute, London, UK

**Keywords:** Yoga, meditation, mindfulness, well-being, complementary medicine, integrative medicine, perceived stress, self-compassion, self-regulation, empathy

## Abstract

**Objective:**

An effective career in medicine requires empathy and compassion, yet the demands of a medical education increase stress and decrease students’ ability to connect with patients. However, research suggests mind-body practices improve psychological well-being. This study aimed to evaluate the psychological effects on medical students of an 11-week elective course, Embodied Health or EH, which combines yoga and meditation with neuroscience didactics.

**Methods:**

The effects on 27 first- and second-year medical students were evaluated via surveys in four areas: empathy, perceived stress, self-regulation, and self-compassion. Scales used were 1. Jefferson Scale of Physician Empathy, which measures empathy among health students and professionals and medical students on a scale of 1 (least empathetic) to 7 (most empathetic); 2. Cohen's Perceived Stress Scale, a measure of the perceived uncontrollability of respondents' lives, from 0 (least stressed) to 4 (most stressed); 3. Self-Regulation Questionnaire, which measures the development and maintenance of planned behavior to achieve goals, from 1 (least self-regulated) to 5 (most self-regulated); and 4. Self-Compassion Scale, which measures self-criticism, from 1 (least self-compassionate) to 5 (most self-compassionate). Students also reflected on EH's impact on their well-being in a post-course essay.

**Results:**

Self-regulation and self-compassion rose 0.13 (SD 0.20, p = 0.003) and 0.28 (SD 0.61, p = 0.04), respectively. Favorable changes were also seen in empathy and perceived stress, which went up by 0.11 (SD 0.50, p = 0.30) and down by 0.05 (SD 0.62, p = 0.70), respectively; these changes did not reach statistical significance. Students’ essays were found to discuss the following recurrent themes: 1) Reconnection between mind and body; 2) Community in a competitive environment; 3) Increased mindfulness; 4) Confidence in use of mind-body skills with patients; and 5) Stress management. These themes overlapped with the measures EH affected quantitatively.

**Conclusion:**

A mind-body course for medical students increased self-regulation and self-compassion. Qualitative themes discussed in students’ post-course essays reflected these effects.

## Introduction

An effective career in medicine requires technical expertise and competency coupled with high levels of empathy, personal well-being, and connectedness to others. In addition, research indicates patients’ perception of physician empathy correlates with levels of satisfaction and compliance with medical recommendations (1). Ironically, medical students report that the demands of medical education – such as a heavy workload, abundant stress, and a sense of competition – tend to increase burnout and decrease the ability to connect with patients (2). For example, overall empathy levels drop as burnout and suicidal ideation temporally rises among students (2).

However, research on yoga and mindfulness meditation suggests these practices may improve psychological well-being, such as by decreasing perceived and physiological measures of stress (3–5). For example, mindfulness-based stress reduction (MBSR), a standardized 8-week program that includes mindfulness meditation and yoga practice, has been shown to increase empathy and reduce anxiety in nursing, medical, and premedical students (4, 6). In addition, research on the Buddhist practice of *metta* meditation – also called loving-kindness meditation – has found that regular practice of this technique can increase social connectedness, positive emotions, self-compassion, and mindfulness, while decreasing stress (5, 7).

Finally, mind–body practices, such as meditation, have been shown to increase self-regulation – the ability to effectively manage one's thoughts and actions to complete a task (8). While an important skill for any person, an increase in self-regulation could prove particularly invaluable for medical students, who face a heavy academic workload and need to rapidly memorize and master a vast amount of information while undergoing frequent testing.

In 2012, Boston University School of Medicine (BUSM) introduced Embodied Health, an elective course combining yoga and mindfulness techniques with an educational component, which focused on scientific research on mind–body practices. Embodied Health was developed with three aims: 1) To provide first- and second-year medical students with a supportive environment in which to learn about yoga, mind – body medicine, and neuroscience through lectures and their own experiences; 2) To improve students’ health and well-being while providing them with information not otherwise covered in medical school; and 3) To teach students practices which ultimately can be taught to their future patients for better health. Here, we present an initial evaluation of the short-term effects of Embodied Health on medical student empathy, self-compassion, self-regulation, and perceived stress.

## Methods

Course enrollment was limited to 27 medical students in their first or second year of medical school at BUSM. Once weekly for 11 weeks over the course of the semester, students learned breathing and meditation exercises and participated in an hour-long yoga session, with the assignment of practicing these techniques at least three times a week as documented by the student. In addition, each class entailed a 30-minute lecture about the neuroscience of yoga, relaxation, and breathing exercises.

### Course development and structure

BUSM offered a yoga and mindfulness elective course, Embodied Health, for the first time in spring 2012. The course was designed and taught by one of the coauthors (Heather Mason) with input from student advisors (Allison Bond, Emily Holick, Caroline Mullin, Stephanie Shaw). The learning objectives were as follows:

1. Promote student wellness by reducing stress, cultivating resiliency to stress, enhancing well-being, and nurturing empathy.
2. Offer a subjective and experiential context for aspects of the medical curriculum, including, but not limited to, neuroscience.
3. Disseminate cutting-edge information on the efficacy of mind – body practices beyond what is available in the current curriculum.
4. Foster student community.
5. Appreciate the relationship between mental and physical health.

The class took place on a weekday evening at the medical school. Enrollment in the elective was completely optional. Students did not receive a grade for their participation; however, as with all electives taken at BUSM, students who successfully completed the course received a note placed in the academic file stating that the student had participated in the extracurricular activity.

To create a personal experience for enrollees, enrollment was limited to 27 first- or second-year students in good academic standing. Elective enrollment was based on a lottery system, as student applicants exceeded the number of places in the course. Students enrolled in the program were given the option to participate in this educational evaluation. Their decision to participate or not in the evaluation had no effect on their enrollment in the elective. The course entailed 11 weeks of 1.5-hour weekly classes.

Each course meeting entailed one hour of deep breathing, meditation, and yoga, and 30 minutes of a neuroscience lecture. In addition, two to three peer-reviewed articles related to the upcoming lecture were emailed to students before each class to further expand students’ knowledge of mind – body medicine. Table 1 shows the course topics and readings by week. The instructor assigned students mind – body ‘homework’ to practice daily at home, such as deep breathing exercises and meditation. Attendance was taken at all classes. To maintain enrollment in the elective, students were not allowed to miss more than one class.

**Table 1:** Course themes and assigned readings

Week	Theme	Reading assigned
1	Introduction	No reading assigned.
2	Effects of slow breath on physiology	Bernardi L, Gabutti A, Porta C, Spicuzza L. Slow breathing reduces chemoreflex response to hypoxia and hypercapnia, and increases baroreflex sensitivity. J Hypertens 2001; 19: 2221–29.
		Joseph CN, Porta C, Casucci G, et al. Slow breathing improves arterial baroreflex sensitivity and decreases blood pressure in essential hypertension. Hypertension. 2005; 46: 714–18.
3	Resistance breathing	Brown RP, Gerbarg PL. Sudarsahn kriya yogic breathing in the treatment of stress, anxiety, and depression: Part I—neurophysiologic model. J Altern Complem Med. 2005; 11: 189–201.
		Jerath R, Edry JW, Barnes VA, Jerath V. Physiology of long pranayamic breathing: Neural respiratory elements may provide a mechanism that explains how slow deep breathing shifts the autonomic nervous system. Med Hypotheses. 2006; 67: 566–71.
4	Yoga and GABA	Streeter CC, Whitfield TH, Owen L, et al. Effects of yoga versus walking on mood, anxiety, and brain GABA levels: A randomized controlled MRS study. J Altern Complem Med. 2010; 16:1–8.
		Streeter CC, Jensen JE, Perlmutter RM, et al. Yoga asana sessions increase brain GABA levels: a pilot study. J Altern Complem Med. 2007; 13:419–26.
5	The relaxation response	Beary JF, Benson H. A simple psychophysiologic technique which elicits the hypometabolic changes of the relaxation response. Psychosom Med. 1974; 36:115–20.
		Dusek JA, Otu HH, Wohlhueter AL, et al. Genomic counter-stress changes induced by the relaxation response. PLoS ONE 3(7):e2576.
6	Lower back pain	Saper RB, Sherman KJ, Cullum-Dugan D, et al. Yoga for chronic low back pain in a predominantly minority population: a randomized controlled trial. Altern Ther Health Med. 2009; 15:18–27.
7	Yoga and PTSD	Emerson D, Sharma R, Chaudhry S, Turner J. Trauma-sensitive yoga: principles, practice and research. Int J Yoga Therap. 2009; 19:123–28.
		van der Kolk BA. Clinical implications of neuroscience research in PTSD. Ann N Y Acad Sci. 2006; 1071:277–93.
8	Mindfulness and chronic pain	Grant JA, Courtemanche J, Duerden EG, Duncan GH, Rainville P. Cortical thickness and pain sensitivity in zen meditators. Emotion. 2010; 10:43–53.
		Kabat-Zinn J. An outpatient program in behavioral medicine for chronic pain patients based on the practice of mindfulness meditation: theoretical considerations and preliminary results. Gen Hosp Psychiatry. 1982; 4:33–47.
		Zeidan F, Martucci KT, Kraft RA, et al. Brain mechanisms supporting the modulation of pain by mindfulness meditation. J Neurosci. 2011; 31:5540-8.
9	Mindfulness for the mind	Hölzel1 BK, Lazar SW, Gard T, et al. How does mindfulness meditation work? Proposing mechanisms of action from a conceptual and neural perspective. Pers Psych Sci. 2011; 6:537–59.
10	The placebo effect	Benedetti F, Pollo A, Lopiano L, et al. Conscious expectation and unconscious conditioning in analgesic, motor and hormonal placebo/nocebo responses. J Neurosci. 2003; 23:4315–23.
		Colloca L, Benedetti F. Placebos and painkillers: Is mind as real as matter? Nat Rev Neurosci. 2005; 6:545–52.
11	Course summary	No reading assigned.

The weekly one-hour experiential section of Embodied Health followed 30 minutes of didactic-style lectures about mind – body research. Next, students were led by a yoga teacher (Heather Mason) through yoga postures (*asana*), breathing techniques (*pranayama* and *ujjayi*), and meditation (*dhanyas*). Certain weeks also included stress management techniques. Students were instructed to observe, but not react to, changing or enduring sensory experiences, such as muscular fatigue or discomfort.

The asana practice differed each week. For example, during the first three weeks, students followed a basic *vinyasa* flow sequence focusing on breath awareness during asana, with pranayama practice in the seated or supine position. From weeks four through nine, students continued to practice slow resistance breathing during asana, but it was not the main focus of the class. Classes included activities such as slow and measured asana practice to calm the nervous system, followed by a 15-minute relaxation-based exercise designed to help students initiate the relaxation response. Other weeks focused on mindfulness of the body from different vantage points; one week's practice ended with a 10-minute guided walking meditation practice.

This educational evaluation was determined to be exempt in accordance with 45 CFR 46.101 by the Boston University Medical Campus Institutional Review Board.

### Data collection

Surveys were administered online using Survey Monkey™ (Portland, Oregon) within the week prior to the commencement of the course, and within one week after course completion. The principal investigator assigned each student a unique confidential study identification number. All researchers other than the principal investigator were blinded to the identity of each survey respondent.

The pre-and post-course online survey included four scales: 1) Jefferson Scale of Physician Empathy, which measures empathy among physicians, health professionals, and medical students on a scale of 1 – 7, with 1 being least empathetic and 7 most empathetic (9); 2) Cohen's Perceived Stress Scale, a measure of how unpredictable and uncontrollable respondents perceive their lives to be, on a scale of 0 – 4, with 0 being least stressed and 4 being most stressed (10); 3) Self-Regulation Questionnaire, which measures the ability to develop, implement, and flexibly maintain planned behavior in order to achieve one's goals, on a scale of 1 – 5, with 1 being least self-regulated and 5 being most self-regulated (11); and 4) Self-Compassion Scale, which measures self-criticism and compassion toward oneself, on a scale of 1 – 5, with 1 being least self-compassionate and 5 being most self-compassionate (12, 13). The Jefferson Physician Empathy Scale was designed for use on medical students and physicians (9). The Perceived Stress Scale and Self-Regulation Questionnaire have been used to evaluate nursing students (14, 15), the Self-Compassion Scale has been used to study social work students (16).

At the conclusion of the elective, students were required to write a one-page essay about their experience in the course. Students were asked to reflect on the following questions: ‘Has this elective or the practices you engaged in as taught by the elective provided you with any personal insights?’ and ‘In what ways, if any, has this elective influenced your ability to cope with stress and/or enhanced your sense of well-being?’ Essays were completed by the final course meeting.

### Data analysis

Summary statistics were used to describe pre- and post-measures. For each participant, a change score for each of the four measures was calculated by subtracting the post-course from the pre-course values. The mean change score was calculated for each measure. A two-sided student *t*-test was used to determine whether the mean change score was different than zero. Data analysis was conducted using Epi Info™ (Centers for Disease Control and Prevention, Atlanta, GA).

In addition, a qualitative evaluation of the students’ reflective essays was completed. Two authors (Allison Bond and Heather Mason) separately analyzed the essays thematically. These two authors, after reading a five-essay sample of the 24 submitted essays, together agreed upon five umbrella themes: 1) influence of the course on school performance; 2) influence of the course on stress management and well-being; 3) influence of the course on perception of medical trajectory; 4) experience of community in the course; and 5) influence of the course on general feelings about mind – body practice. Both authors read the essays separately, keeping a tally regarding how many essays discussed a given theme. Subsequently, the authors compared findings, resolving differences by discussion.

## Results

Twenty-seven students were enrolled in the course out of a total of 32 who applied for the class. All enrolled students completed the pre-course survey; 24 filled out the post-course survey. The 27 students attended a median of 11 classes (range 9 – 11). Of the 27 course participants, 15 were first-year and 12 were second-year medical students.

The participants’ mean perceived stress score decreased from 1.55 to 1.48 during the course (p = 0.70). Mean self-regulation increased a statistically significant amount from 3.49 to 3.58 (p = 0.003). Total self-regulation scores, which are used to gauge overall self-regulation scores as stated by the scale, were 219.9 and 225.5 before and after the course, respectively. Self-compassion also increased by a statistically significant amount: mean baseline value was 2.88 compared to 3.25 post-course (p = 0.04). Empathy levels also increased, but this effect was not statistically significant. Initial empathy scores averaged 5.64, while final scores averaged 5.80 (p = 0.30). Table 2 shows mean change scores and effect sizes for each measure.

**Table 2:** Mean change in scores for different measures

Measure	Mean change score	SD	p	Cohen's d
Cohen's Perceived Stress Scale	−0.05	0.62	0.70	0.14
Self-Regulation Questionnaire	0.13	0.20	0.003	−0.41
Jefferson's Scale of Physician Empathy	0.11	0.50	0.30	−0.31
Self-Compassion Scale	0.28	0.61	0.04	−0.55

Twenty-four students wrote post-course reflective essays. Table 3 lists the five common themes identified and the number of essays that discussed each theme. Here, we provide a description of each theme with illustrative quotations from participants:

**Table 3:** Theme and number of essays mentioning the theme

Theme	Number of essays (%)
Reconnection between mind and body	18 (75)
Community in a competitive environment	4 (17)
Positive effects of increased mindfulness	16 (67)
Increased ability to share CAM with patients	19 (79)
Increased ability to manage stress	19 (79)

CAM, complementary and alternative medicine.

### Reconnection between mind and body

Many students cited an increased awareness of the body and its responses to various sensations and situations. Students were able to use this awareness to modulate physiological responses.

During each yoga practice, we were encouraged to take detailed mental snapshots of our physical states during different stages of the practice … As the semester went on, the individual temporal snapshots turned into a seamless ability to self-monitor and subsequently self-regulate … I learned to immediately detect when my body felt different in any way and I could thus modify my actions accordingly. I took this new skill with me to my daily activities: I could recognize when I was slouching and thus fix my posture accordingly. I could tell when my shoulders were tense and thus relax them accordingly.

Some students also discussed a newfound awareness of physical sensations and the emotions they may bring without feeling the desire to change the sensations or feelings.

For the first time in years, I was ready to listen without resentment, to acknowledge with acceptance what my body was trying to tell me.

### Creating community in a competitive environment

Participants noted an increased sense of camaraderie between students in the course. Medical school can be an isolating experience for students, who may believe they are the only ones having difficulty coping with its extreme demands. In Embodied Health, some students stated they gained a sense of community by doing yoga with others going through the same struggles and stressors. Finally, students noted increased self-awareness, as well as newfound self-compassion to accept oneself and these thoughts.

I really liked that I was taking this class with other medical students. I have taken yoga classes in the past that were open to the public, and while I enjoyed them there was something unique about taking a class with fellow medical students. Since we take the same courses and go through the ‘medical school experience’ together, it's easier to talk to each other and share in the experience.

In addition, some mentioned becoming aware of internal conflicts and subconscious self-judgments.

I did not realize how competitive I was, or [how] prone to compare myself to my peers … I did notice that I tend to judge myself harshly and compare what I am doing to other people, feeling confident when I am doing more than the majority. Now that I am more conscious of that thought process in my life, I can work on unraveling it and replacing it with other ways to motivate myself.

This year was a really hard year for me, not only because of the pressure of school but because of other issues in my life. Having a place to come every week, a community, where I felt connected was wonderful. Also, I never imagined that in the competitive environment of medical school other students were scared or struggling. It gave me solace to know that I was not alone.

### Increased mindfulness

Mindfulness – keeping one's thoughts ‘present’ in the current moment, activity, or location, while remaining non-judgmental about sensations and feelings – was another common theme in the essays. Participants expressed increased physical and emotional health outside of school due to an improved ability to manage stress.

The yoga was therapeutic in helping to remind me to let go of the things that I cannot control in my life. I was able to cope with a stressful situation without feeling like there was something else I needed to try to do to change it … I was reminded that sometimes the best thing to do is to breathe, to be present in the moment, to listen to my body, and to accept what is happening.

Others cited specific examples of physical problems that were alleviated by an increased awareness of thoughts and physical sensation as a result of the class.

After discovering that paying attention to my breathing helped me deal with stress very efficiently, I … think that my sleeping habits have improved because of my ability to focus on my breathing or on how one part of my body specifically feels.

### Confidence in using mind – body skills with patients

Many essays discussed an evolution in students’ future plans for patient care. Participants cited increased confidence in instructing or referring patients to mind – body techniques, such as mindfulness meditation, breathing exercises, and basic yoga poses. Through reviewing the scientific literature, students expressed increased knowledge and understanding of the evidence supporting the physical, mental, and emotional benefits of mind – body practices.

If I recommend yoga to a patient with anxiety and follow-up with the patient two weeks later, and she says that she keeps looking at the other people in the class and feels like she should try to keep up with what they are doing, then I will be able to reflect on my own experience to comfort her … I would not understand what feeling she is describing or how to advise and encourage this future patient without understanding the practice myself.

The combination of learning the techniques and learning about the scientific evidence of their benefit has been incredible. It has allowed me to grasp these concepts in a personal/spiritual way as well as a rational/scientific way, making it extremely memorable. I now feel much more confident that I could recommend yoga to family, friends, and my future patients!

My perspective on medicine has changed since being a part of Embodied Health. I now realize how valuable yoga and other alternative treatments can be in treating chronic pain, post-traumatic stress disorder, anxiety, and depression. I think it is essential that physicians present all of the treatment options to their patients.

By coming to not only learn about, but experience practices revolving around yoga and mind – body awareness, our abilities as providers to help our patients increases exponentially. By increasing the tools available to us, we are able to expand our roles beyond that of medical providers, and instead emerge as practitioners of health: a type of health defined by far more than the simple absence of disease.

### Stress management

Essays described better management of stress during the class itself, and by using techniques learned in the course while in the home or classroom setting.

Before this class began, I had an anxiety problem … My heart would start beating really fast, and I couldn't breathe well. This even occurred when I returned from break and attempted to go to sleep … When I learned the breathing exercise in the first class (with the bells/chimes), I immediately felt more at ease. By the third week of class, my symptoms had disappeared, and I have not experienced them since.

Any specific thought that would generate stress became an opportunity to focus on the slice of my life it had originated from and, as before, modify it accordingly.

Other students discussed decreasing stress through mindfulness, making it possible to fully relax in students’ limited free time.

As a medical student, I am constantly thinking of problems and solutions. My mind feels like it is running a marathon, but I am trying to sprint the entire race. It was very difficult to shut off my mind during yoga practice for the first couple of weeks. By the last week, I felt that I had created some space in my mind.

## Discussion

A semester-long mind – body course for medical students had a statistically significant positive effect on students’ self-regulation and self-compassion. There was also a favorable change in empathy and perceived stress, but these changes did not reach statistical significance. Our study also suggests there are subjective benefits to Embodied Health which overlap to a great extent with the psychological measures we measured, particularly self-regulation and self-compassion.

Students’ self-regulation scores before and after the course fall within the ‘moderate’ self-regulation capacity range. Regarding self-compassion, previous studies have found mean scores for undergraduates to be 3.04 (11). Thus, our study showed students initially tended to be less self-compassionate than the average undergraduate student, whereas after the course, their self-compassion was greater than average. Previous research has found the average empathy score among first-year medical students to be 5.93, whereas it was 5.91 among second-year medical students (17). Thus, we found that students in the course had lower empathy scores both before and after the course than previously published data on medical students.

Students’ baseline perceived stress scores indicate that medical students are more stressed than their gender- or age-matched peers. The mean population-based perceived stress scale, based on a 1988 national area-probability sample of 2,387 non-institutionalized adults in the United States, was found to be 1.21 on a scale of 0 – 4 (18). The same study found the average for women was 1.37 (18). In addition, the national average among 645 test-takers aged 18 – 29 was found to be 1.42 (18).

Common themes found in students’ end-of-course essays included increased mindfulness and its positive effect on self-regulation; a feeling of community in an otherwise competitive environment with an increase in empathy for others; positive effects of increased mindfulness as reflected by increased self-regulation; a decrease in stress level; and increased competency and interest in suggesting mind – body practices that could benefit patients. These themes overlapped significantly with the measures that were affected by the course – that is, self-regulation and self-compassion.

Previous studies have shown yoga and other mind – body practices, such as MBSR, increase self-regulation and self-compassion. However, the subjects of these studies were not medical students (7, 8). Rosenzweig et al. examined the effects of MBSR in medical students and found decreased tension, anxiety, and total mood disturbance compared to a control group attending didactic seminars on complementary medicine (19). Other research found that an eight-week meditation-based course decreased stress and increased empathy levels in premedical and medical students (20).

Contrary to our results, some research has shown yoga to reduce perceived stress among medical students and in non-medical student populations (19–21). For example, a three-month intensive yoga program was shown to decrease stress among distressed women (21). However, the study entailed twice-weekly 90-min Iyengar yoga classes. Thus, our program's one hour of yoga weekly may have been too little to significantly reduce stress. It is also possible that any potential stress-alleviating benefits were offset by the escalating stress over the course of the spring semester, during which there is an increasingly heavy academic load for both first- and second-year students. In particular, students in their second year face the impending United States Medical Licensing Exam – Step 1, a stressful exam considered highly important for residency applications. However, without a control group, determination of any specific effect by Embodied Health is not possible.

BUSM's Embodied Health course is among only a few in the United States teaching medical students yoga and mind – body medicine both didactically and experientially (22). Participant knowledge of the science behind mind – body practice, not typically taught as part of the medical school curriculum, is enhanced. It also offers opportunities for students to learn new skills that they can pass on to their future patients. One other mind – body course for medical students found five central themes to students’ responses to the course – connections, self-discovery, stress relief, learning, and medical education (22). A mindfulness program for primary care physicians has also been found to improve measures of psychological well-being, such as by improving mood and decreasing burnout (23). In addition, given that almost 40% of US adults reported in 2007 that they had used some form of complementary or alternative therapies within the past year, scientific and experiential knowledge of these practices is valuable to students’ future capacity to counsel patients on the appropriate use of complementary therapies (24).

There are several important limitations to this research. First, there was no control group. Without a control group, it is not possible to ascertain whether observed changes were due to the course or to other factors. In addition, a small sample size limited the statistical power of the study. Students in the study were fairly high in empathy at baseline, suggesting a possible ceiling effect for this measure. In addition, we lack long-term follow-up data regarding students’ psychological well-being, as the study was short-term (one semester) only. Another limitation of the study is generalizability; students enrolled themselves in the study and course voluntarily and were therefore self-selected. Thus, students taking the course may have possessed certain interests and traits that other students do not.

Future studies should examine the long-term effects of a mind – body practice on medical student well-being, even after finishing medical school and entering residency and clinical practice. In addition, larger and longer studies are needed to better assess the quantitative effects of a mind – body course on medical students.
